# 982. Microbiologic Composition and Failure Rates of Prosthetic Hip and Knee Infections Managed with Debridement, Antibiotics and Implant Retention.

**DOI:** 10.1093/ofid/ofac492.824

**Published:** 2022-12-15

**Authors:** Joseph M DeBiase, Shandra R Day, Jessica M Smith, Sydney Agnello

**Affiliations:** The Ohio State University Wexner Medical Center, Columbus, Ohio; Ohio State University Wexner Medical Center, Columbus, Ohio; The Ohio State University Wexner Medical Center, Columbus, Ohio; The Ohio State University, Columbus, Ohio

## Abstract

**Background:**

Success rates of prosthetic joint infections (PJI) managed by debridement, antibiotics, and implant retention (DAIR) vary according to recent literature. Our aim is to evaluate the success/failure rate of PJI managed by DAIR with respect to the microbiologic composition, antimicrobial selection, and treatment duration.

**Methods:**

A single-center retrospective cross-sectional study was conducted from January 2017 to December 2021. All patients ≥ 18 years of age who underwent DAIR were identified from an existing internal database. Patients were included if they received at least 3 weeks but no more than 8 weeks of parenteral therapy with or without consolidative or chronic therapy. Data collection was completed via chart review including demographics, microbiologic data, antimicrobial therapy and outcomes. Failure was defined as any subsequent need for surgical intervention, infection related mortality, persistent infection, new infection, or probable treatment failure measured at a minimum of two year follow up from index DAIR.

**Results:**

A total of 49 patients were included (15 hip, 34 knee) with 21 failures (43%) and 28 in remission (57%). Table 1 shows the breakdown of cases including time to PJI from index procedure, duration of symptoms prior to DAIR procedure, chronic antibiotics, and microbiologic composition. DAIR procedure occurred < 3 weeks from onset of symptoms in 43 patients (88%) but no observed difference in outcomes was seen based on patient’s time to PJI or symptoms duration. *Staphylococcus aureus* was the most common pathogen identified, n=14 (29%) and encompassed 43% of the total failures. Mean antimicrobial therapy was 42 and 180 days for parenteral and oral respectively with 17 (35%) patients on chronic/indefinite therapy. Of the failures, 48% occurred while on parenteral therapy. Need for further surgical intervention was the leading cause of failure at 37%.

**Figure:**
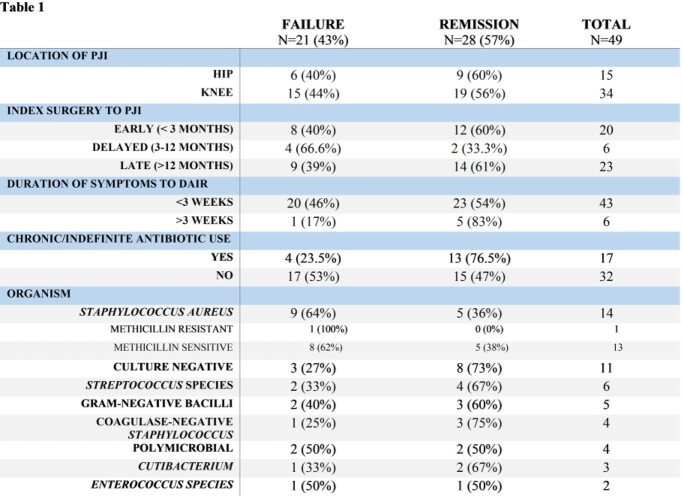

**Conclusion:**

Despite early intervention, our study found a failure rate of 43% in patients with knee and hip PJI managed with DAIR. We observed similar success rates across multiple organisms and antimicrobial treatments. Further evaluation of failure cases would help to inform which patients are the best candidates for DAIR procedures.

**Disclosures:**

**All Authors**: No reported disclosures.

